# A Multicentre Study of the Attitude of Medical Students towards Organ Donation and Transplantation in Poland

**DOI:** 10.3390/ijerph20043711

**Published:** 2023-02-20

**Authors:** Marzena Mikla, Anna Maria Cybulska, Daria Schneider-Matyka, Antonio Ríos, Mariusz Panczyk, Artur Kotwas, Beata Karakiewicz, Elżbieta Grochans

**Affiliations:** 1 Faculty of Nursing, University of Murcia, 30100 Murcia, Spain; 2 Biomedical Research Institute of Murcia (IMIB), 30120 Murcia, Spain; 3 Department of Nursing, Pomeranian Medical University, Żołnierska 48, 71-210 Szczecin, Poland; 4 Department of Surgery, Paediatrics, Obstetrics and Gynaecology, University of Murcia, 30100 Murcia, Spain; 5 Transplant Unit, Surgery Service, IMIB–Hospital Clínico Universitario Virgen de la Arrixaca, 30100 Murcia, Spain; 6 Department of Education and Research of Health Sciences, Faculty of Health Sciences, Medical University of Warsaw, Litewska 14/16 St., 00-518 Warsaw, Poland; 7 Subdepartment of Social Medicine and Public Health, Department of Social Medicine, Pomeranian Medical University in Szczecin, 70-213 Szczecin, Poland

**Keywords:** transplantation, students, attitude towards organ donation, transplantation

## Abstract

(1) The aim of the present study was to assess the effect of sociodemographic (age, sex, religion, place of residence) and university-related factors (university, year of studies) on the attitudes of students towards organ donation and transplantation. (2) Methods: The study was conducted on 1530 students from the Faculty of Medicine from three medical universities in Poland. The measurement tool was a validated questionnaire of attitude towards organ donation and transplantation (PCID-DTO RIOS: A questionnaire designed by the International Collaborative Organ Donation project about organ transplantation and donation). (3) Results: The completion rate was 88.10% (n = 1348). The vast majority declared a willingness to donate their organs in the future (86.60%), and 31.71% had an organ donation card. It was found that place of residence (*p* = 0.018) and religion (*p* = 0.003) had a significant effect on the attitude towards transplantation. Age, sex, and year of the study were not found to have a statistically significant effect on the decision. (4) Conclusions: The present study demonstrates that medical students show a favourable attitude towards transplantation in the first year of their study, and their knowledge and positive tendencies increase in the final years of medical education.

## 1. Introduction

In cases of end-stage organ failure, organ transplantation is the best and often the only lifesaving treatment option that may noticeably improve the quality of life and life expectancy. Organs and tissues for transplantation can be obtained from living or deceased donors [1]. Despite the advances regarding organ donation and transplantation, the current donation rates remain insufficient to meet the minimum needs. Organ shortage is the main cause of the death of patients on the transplant waiting list. According to the 2020 data of the WHO Global Observatory on Donation and Transplantation, the number of solid organ transplantations worldwide exceeds 130,000, which probably accounts for 10% of the worldwide demand. In Poland in 2021, there were 1335 organ transplantations, 770 of which were kidney transplants (726 from deceased and 44 from living donors), 296 liver transplants (277 from deceased and 20 from living donors), 200 heart transplants, 68 lung transplants, and 20 pancreas transplants [1].

The organ donation rate for a region is calculated as the number of organ donors per million population (PMP). According to the 2021 report by the Global Observatory on Donation and Transplantation, the countries with the highest organ donation rates are, respectively: USA (41.6 PMP), Spain (40.8 PMP), and Iceland (36.7 PMP). For Poland, the rate is 10.5 [1]. The report points to a significant decrease in the number of transplantations, particularly in 2020, mainly due to the COVID-19 pandemic.

Transplantation activity is unsatisfactory, even in the countries considered as advanced with respect to organ transplantation. There are numerous factors affecting organ donation [2]. Sociocultural factors are one of the main sources of the variability identified in the studies on attitudes towards organ donation [3]. Additionally, the geographical area can influence the willingness to donate organs. Individuals from similar cultural backgrounds living in different geographical areas may show different attitudes towards organ transplantation. Additionally, the concept of death, religious beliefs, or sociodemographic factors (age, sex, level of education) have been found to significantly affect the attitudes towards organ donation and transplantation [4].

The public should be informed about the possibility of donating organs for transplantation. In this respect, of particular importance is the role of the physician who identifies the potential donors, communicates with the organ donation coordinator, or obtains the consent from the family of the potential donor. Therefore, it is vital that health care workers have sufficient knowledge and exhibit greater awareness regarding organ donation and transplantation than that found in other social and professional groups.

Health care workers play an important part in the successful organ donation process [5]. In turn, medical students are the new generation of clinicians who, in future, will act as a link between the donors and recipients. Consequently, obtaining knowledge on the attitudes of medical students concerning organ donation is considered a particularly important issue that has been investigated in the literature. Studies have been conducted in various countries, cultural, and religious backgrounds. The analyses concerned different dimensions of organ donation attitudes and used various methodological procedures. The obtained data indicate a significant gap in understanding the key factors that could markedly affect the attitudes of future health care workers concerning organ transplant including xenotransplantation [6]. For this reason, the aim of the authors of the present paper was the assessment of the effect of the sociodemographic (age, sex, religion, place of residence) and university-related factors (university, year of study) on the attitudes of medical students concerning organ donation and transplantation.

## 2. Materials and Methods

### 2.1. Settings and Design

**Figure 1:**
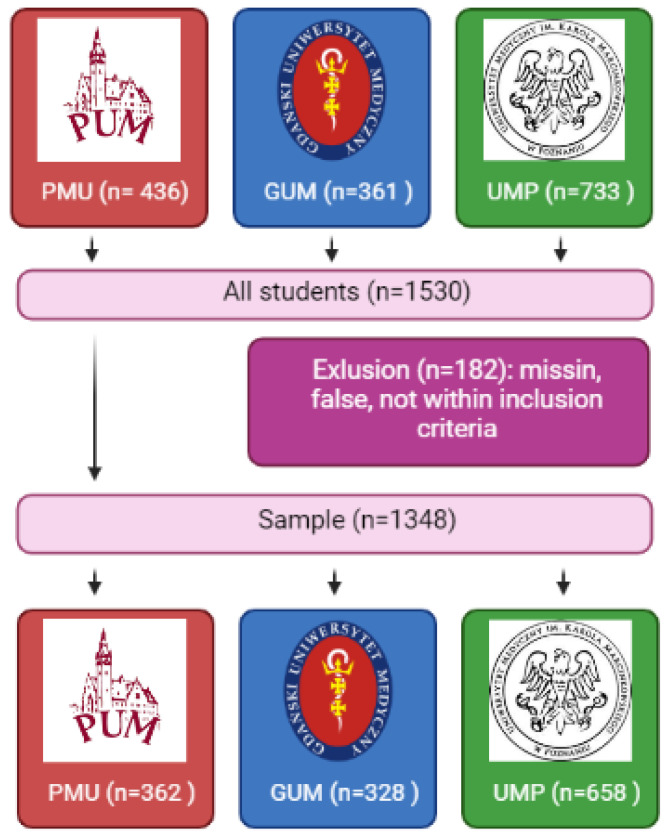
Selection of the study group.

The study was conducted in 2022 on 1530 students from the Faculty of Medicine at three medical universities in Poland: Pomeranian Medical University in Szczecin (PMU), Poznań University of Medical Sciences (UMP), and Medical University of Gdańsk (GUM) (Figure 1).

The inclusion criteria were as follows: Age over 18 years, being a student of the Faculty of Medicine at one of the three medical universities in Poland (Pomeranian Medical University in Szczecin, Poznań University of Medical Sciences, and Medical University of Gdańsk) and informed written consent to participate in the study.

The study was conducted using the diagnostic survey method with a questionnaire technique. The research procedure was conducted in accordance with the Declaration of Helsinki following the approval by the Bioethical Committee (KB-012/260/06/2016). The respondents were informed about the aim of the study, the possibility of withdrawing consent at any time, and were given the opportunity to ask questions and obtain a thorough explanation. The respondents who gave their written consent were given a survey questionnaire for self-administration.

### 2.2. Research Instruments

The study was made using the standardised validated questionnaire of attitude towards organ donation and transplantation [PCID-DTO RIOS: A questionnaire designed by the ‘International Collaborative Organ Donation project about organ transplantation and donation (‘Proyecto Colaborativo Internacional Donante sobre la Donación y Transplante de Organos’ in Spanish) developed by Dr Ríos] (Ríos et al. 2006a, 2007a, 2008, 2010a, 2014, 2015) [7]. The validation process consisted of two parts: the translation and evaluation of the psychometric properties of the instrument translated into the Polish language.

As dependent variables, study attitude towards organ donation themselves, the independent variables to study, were (1) personal-social; (2) information and knowledge about organ donation and transplantation (ODT); (3) social interaction; (4) prosocial behaviour; (5) religion; and (6) attitude towards the body. Cronbach’s alpha reliability coefficient for the Spanish population was 0.834.

Out of the 1530 medical students invited to participate in the study, 1348 students who met all of the inclusion criteria and completed the questionnaire correctly were enrolled in the study (completion rate: 88.10%).

### 2.3. Statistical Analysis

Quantitative and categorical variables were described with descriptive statistics methods. For the quantitative variables, the following measures were determined: central tendency (mean, M) and dispersion (standard deviation, SD). For the categorical variables, the following measures were determined: number (N) and frequency (%).

All of the calculations were performed with STATISTICATM 13.3 software (TIBCO Software, Palo Alto, CA, USA). For all of the analyses, a p-level of <0.05 was considered statistically significant [8].

## 3. Results

### 3.1. Characteristics of Respondents

The study group comprised 1348 medical students, with a mean age of 22.07 (SD = 2.13). The majority of respondents were female (61%), students of Poznań University of Medical Sciences (49%) in the second year of their studies (35%), and residents of a city with more than a population of 100,000 (73%) (Table 1).

### 3.2. Assessment of the Attitude towards Organ Transplantation

The analysis concerned the questions regarding their opinion on organ donation, general knowledge about transplantology, willingness to donate organs for a non-relatives or a family member, willingness to be a recipient of organ donation, and communicating the possibility of donating organs. Table 2 presents the collected data as the opinions of medical students on selected aspects of organ donation.

The students from the Faculty of Medicine at three Polish universities reported that in the vast majority of cases, none of their family members needed an organ transplant (95%), nor was an organ donor (95%). Almost all students (97%) did not agree with the opinion that the number of organ donors is sufficient to meet the demand. A total of 74% responded positively to the question of whether they were informed about the possibility of donating organs. Moreover, 97% of the respondents would agree to organ donation on behalf of a family member. A definite majority of students discussed the possibility of donating their organs to family members (58%) or friends (73%), and declared their willingness to donate one’s organs in the future (73%). Among the study group, 31% had an organ donation card (Table 2).

### 3.3. Reasons for Consent or Refusal to Donate Organs in Future

The overwhelming majority of respondents did not donate blood (71%), and 7% did it regularly. Among the most frequently stated reasons for consent to organ donation were solidarity (67%) and moral duty (50%). In turn, in the opinion of 33 respondents, fear of organ removal before death had an influence on organ donation refusal. For 12 respondents, the decision on organ donation was affected by the awareness of the manipulation of the body after death.

The medical students under study were asked for their opinions of their relatives concerning organ transplantation. It was found that 33% of fathers, 47% of mothers, and 31% of partners of the respondents declared being in favour of organ transplantation (Table 3).

The most frequently reported sources of positive opinions regarding organ transplantation given by the students at medical universities were the Internet (77%), books and brochures (71%), and school (69%) (Table S1).

### 3.4. Opinion about Donating One’s Organs after Death vs. Sociodemographic Factors and Religion

The study involved the analysis of the effect of sociodemographic factors (age, sex, religion, place of residence) and university-related factors (university, year of study) on the opinions concerning organ donation and transplantation.

The analysis of the collected data showed that the place of residence and religion had a statistically significant effect on the answer to the question “Would you consider donating your organs after death?” It was found that the residents of large cities (over 10,000 residents) (*p* = 0.018) and atheists or agnostics (*p* = 0.003) expressed the slightest doubt concerning organ donation. The remaining variables (age, sex, year of studies, university) had no statistically significant effect on their decision (Table 4).

The analysis of the collected data showed that both the decision of donating one kidney (to a non-relative and to a family member) as well as the opinion about the risk connected with living kidney donation was statistically significantly dependent on the possibility of donating one’s organs after death (Table 5).

The majority of students (92%) who declared a positive attitude towards donating one’s kidney considered the possibility of donating one’s organs after death. In turn, 76% of the respondents who declared the refusal to donate one’s kidney expressed their willingness to donate organs after death (*p* < 0.001).

It was found that 91% of the respondents who considered that there was no great risk in living kidney donation expressed a positive attitude towards donating one’s organs after death. In turn, 17% of those surveyed who had no knowledge on the risk of living kidney donation expressed significant doubts about donating organs after death (*p* < 0.001).

The study showed that 89% of medical students declared their willingness to donate an organ to their family member. However, 24% of the respondents who had doubts about donating one’s organ to a family member expressed doubts about organ donation after death (*p* < 0.001) (Table 5).

According to the analysis of the collected data, the decision on donating a part of the liver (to a non-relative and to a family member) as well as the consent to receive a liver part from a family member together with the opinion about the risk of being a living donor of a part of the liver was statistically significantly dependent on the possibility of donating one’s organs after death (Table 6).

The vast majority of students (92%) who declared a positive attitude towards the possibility of donating a part of their liver considered donating their kidney after death. In turn, 82% who were unfavourable to living liver donation, expressed their willingness to donate organs after death (*p* < 0.01).

It was found that 91% of the respondents who considered living liver donation as partly risky, had a positive attitude towards donating their organs after death. Additionally, 80% of the respondents who believed that the risk of being a living donor of a part of the liver was very high considered organ donation after death (*p* = 0.016).

The study demonstrated that 89% of the respondents would donate a part of their liver to a family member and declared the possibility of donating one’s organs in the future. However, 55% of those surveyed who would not donate a part of their liver to their next of kin expressed a positive attitude towards organ donation after death (*p* < 0.001).

The overwhelming majority of the respondents (90%) who declared that when in need of a liver transplant would consent to receive the liver from a family member, also declared the possibility of donating one’s organs after death.

## 4. Discussion

### 4.1. Consent to Being a Donor

A positive attitude of medical students towards organ donation and transplantation and their future function as a role model for patients are of key importance in convincing patients and their relatives. Therefore, identifying the opinions of medical students regarding organ donation is crucial.

The present study showed that most of the respondents would consent to receive an organ from a family member. A large proportion of students discussed the possibility of donating their organs to family members and friends. The majority of students declared the possibility of organ donation in the future. Additionally, one third of students held an organ donor card. On the grounds of the meta-analysis by Iniesta-Sepúlveda M et al. [6], it was demonstrated that approx. 70% students was willing to donate organs after death.

Numerous factors affect the decision on organ donation in the future such as the geographical area (the continent) or cultural background, among others. The studies concerning this issue were mostly conducted in Latin (82%), Western (70.6%), followed by Muslim (57.7%) and Oriental cultural backgrounds (54.4%), the latter showing the lowest figures. The meta-analysis by Mekkodathil et al. [9] concerning the public opinion in Muslim countries revealed that the total percentage of individuals in favour of organ donation was less than 50%. The analysis of the population of Asia by Li et al. [10] identified a reduced ratio in terms of the willingness to donate organs and register as organ donors among students, health care workers, and the general population. However, the study by Tackmann et al. [11] demonstrated that active and passive acceptance for post-mortem organ donation was high among health care students and trainees in Germany. Most of the surveyed individuals held a written declaration of intent and had it documented in their electronic health card. Most would also agree to organ donation on behalf of their family members. Additionally, Terbonssen et al. [12] identified a significant willingness on the part of medical students to document their intention regarding organ donation after death in the form of the organ donation card. The studies by Ozturk et al. [2] found that the majority of students believed that organ transplant saves lives, and that 73.4% of students considered organ donation. The willingness to donate organs identified by Bilgel et al. [13] amounted to 58.4%. In turn, Naçar et al. [14] found that the differences in terms of the willingness to donate organs in the future were dependent on the year of study and amounted to 45.4% (first year) and 58.8% (sixth year), respectively. Similar results were obtained by Akkas et al. [15], who reported a willingness indicator at 40% (first year) and 60% (sixth year), respectively. The literature review showed a significant differences in terms of willingness to donate organs between medical students in different countries [16].

### 4.2. Factors Affecting the Decision on Organ Donation

The review of the literature on the subject indicates that there are many factors that could significantly affect the decision on potential organ donation [17]. Therefore, given the vast differences in willingness indicator between different countries and social groups, the researchers decided to conduct an assessment regarding the way in which socio-cultural background affects the decision-making process regarding organ donation. Indeed, it was found that many students who refused to donate organs after death could not state the reason for their unfavourable attitude.

However, according to the present study, students who declared unfavourable attitude towards transplantation stated post-mortem body mutilation as the reason. As far as medical students in Muslim countries are concerned, the decision regarding organ donation is determined by the importance of body preservation after death [18]. The studies conducted in Iran revealed that the main reasons for negative attitude towards donating their organs were their religious beliefs and the idea of the violation of the body’s integrity [19]. In turn, the percentage of students concerned with post-mortem body mutilation was found to be markedly lower in Western [16] and Latin [19] cultural backgrounds. The study by Rios et al. [20] demonstrated that individuals accepting body mutilation when in need of transplantation (OR = 2.958) were much more willing to donate organs. According to the studies by Burra et al. [17], medical students are against organ donation mainly because of their reluctance to accept the validity of the adopted brain death criteria and does not consider them to be philosophical, moral, or ethical.

Numerous studies have reported the significant effect of religion and individual attitudes of the respondents on their decisions concerning organ donation. Our own studies have shown that the religion of the respondents had a statistically significant effect on the decision to donate organs in future, whereas agnostics and atheists showed a greater willingness to be an organ donor. This is in line with the studies by Rios et al. [20] (OR = 1.766). Ozturk et al. [2] observed that 50.4% of the surveyed students claimed that their religious beliefs had no negative effect on their attitude towards organ transplantation. The literature review, however, indicates that attitudes and religious beliefs affect the decisions concerning organ transplant among medical students [20]. Tumin et al. [21] noted that medical students whose religion allows organ transplant expressed favourable opinions on organ donation. The studies conducted in Saudi Arabia, Turkey, and Iran showed that some students ignored the fact of whether their religion allows organ donation and transplantation [18,22]. In turn, since the introduction of the Islamic concept of allowing organ donation to a wider group of recipients, a marked increase in the willingness indicator has been observed [23].

However, another important factor affecting the decision to donate organs among medical students was the opinions of their family members concerning transplantation. Additionally, the role of the family in the decision-making process in terms of transplantation can be considered as controversial, for example, in Poland, the system of organ procurement operates under a model of presumed consent with an active objection of the relatives. Our own studies identified that 33.31% of fathers, 47.03% of mothers, and 31.16% of partners of the respondents declared a positive attitude towards organ transplantation. Rios et al. [20] reported that a positive opinion of the father (OR = 1.841), the mother (OR = 2.538), and the partner (OR = 2.192) concerning organ donation had a positive effect on the respondents’ decision regarding transplantation. According to De Ohwaki et al. [24], more than 65% of medical students declared that their family members would not consent to organ donation. The studies by Rydzewska et al. [25] found that 26.4% of students believed that family and friends should have a deciding vote in the process of organ procurement. Trompeta et al. [26] suggested that discussion among family members was a factor that significantly affected the decision to donate organs. This was confirmed by Shumin et al. [27], who emphasised the importance of discussing the issue with family members who were often against post-mortem organ donation as they did not know the intention of their relative in a state of brain death. In turn, Smith et al. [28] demonstrated that the willingness to donate organs increased from 47% to 93%, provided that the family was aware of their relative’s intention. Significant differences observed in the cultures of the East stem from traditional values that place emphasis on family interest rather than that of an individual [29]. In contrast, in Western culture, the opinion of the family had a positive effect on the willingness to donate organs and the share of students discussing organ transplants with their families was found to be significantly higher [25,30]. Moreover, it was found that in Western countries, a higher proportion of medical students believed their parents’ opinion to be favourable [16]. The studies by Ozturk et al. [2] revealed that a markedly increased number of students were willing to donate their organs if their family members were also organ donors. This is in line with the results presented by Rios et al. [20].

The present study did not show a relationship between age and the possibility of donating one’s organs. The meta-analysis by Iniesta-Sepúlveda M et al. [6] demonstrated that age was positively correlated with the percentage of students having a favourable opinion on organ donation. However, it is worth noting that the said relationship may be due to the changing level of education rather than the age of the respondents, given the significant differences between the willingness reported by the students in the first and in the sixth year of study. This was also shown in the studies by Rios et al. [20]. In conclusion, both the appropriate training acquired while studying as well as contact with transplant patients or donors, may have a positive effect on attitudes towards organ donation [16].

The present study did not reveal a relationship between sex and the percentage of students in favour of transplantation, which is consistent with the results by Iniesta-Sepúlveda et al. [6] or El-Agroudy A et al. [31]. However, there have been studies reporting that women show greater willingness towards organ donation than men [2,13,17,32].

It was demonstrated herein that the respondents would consent to receiving an organ from a family member. In turn, as presented by Ozturk et al. [2], 83.8% of the respondents provided an affirmative answer to the question of whether they or members of their immediate family would be willing to receive an organ from another person when in need. Similar results were obtained by Gizibara et al. [33], who showed that over 85% of the surveyed students reported that, when in need, they would consent to receiving organs or tissue from another donor. In addition, it is worth noting that students demonstrated a humanistic approach to organ donation, as most stated that the issue of the identity of the recipient was in fact irrelevant, and they were willing to donate organs to any individual and not just to a family member [16]. Nevertheless, some studies indicated that most students would first donate organs to their family members, relatives, or friends [19].

The present study conducted on medical students indicates that the most frequently reported sources of positive information on organ transplant were the Internet, books and brochures, and school. Most studies reported that the main source of information on organ donation and transplantation was family and friends [2,34,35] as well as educational programmes [36]. Moreover, it was found that students who had knowledge of organ donation and transplantation were more interested in donating their organs [33].

In conclusion, it transpires that despite the awareness and favourable attitude towards organ donation and transplantation among medical students, the number of students registered as organ donors was found to be low. It is therefore suggested that efficient methods such as training or courses are implemented, in order to change the students’ attitude towards organ donation.

## 5. Strengths and Limitations

The study’s strengths lay in the large group of respondents and the use of the standardised questionnaire in order to survey opinions on organ donation and transplantation.

There were some limitations to the present study. The group of respondents should be expanded, taking into consideration other medical universities. Additionally, it would be advisable to consider in the analysis the differences in the curriculum with respect to the issue of transplantation when comparing the opinion of students from different universities.

Among the students who participated in the study, the group of students in their 1st–3rd years was larger than the number of students in their 4th–6th years. This is related to the actual number of students in the particular years of study.

## 6. Implication

According to the present study, students from the Faculty of Medicine were very willing to donate one’s organs; additionally their knowledge and attitude towards transplantation increased with successive stages of education. However, the percentage of students who were against and undecided was still significant. Therefore, it seems essential to improve the educational programme on transplant procedures to encompass the socio-cultural and religious aspects and, consequently, to raise the awareness of future health care workers in this respect. Moreover, the issue of transplantation including organ donation should be addressed in the early stages of education as it may have a positive effect on broadening the understanding of the issue by students as well as their social environment. Increased awareness of students with respect to organ donation and transplantation may make them more inclined to discuss the issue with a wider group of family members or friends.

## 7. Conclusions

The present study demonstrated that students from the Faculty of Medicine had a favourable attitude towards organ donation and transplantation starting from the first year of their study, and their knowledge and positive tendencies increased with higher educational attainment in the last year of their study. Nevertheless, some students did express a negative attitude towards transplantation. Studies investigating the reasons why some students are against transplantation should be continued to influence the opinions of young people starting in their very first year of study.

## Figures and Tables

**Table 1:** Socioeconomic data of the respondents.

Variables (N = 1348)		n	%
Sex	Female	823	61
	Male	525	39
Place of residence	Countryside	118	9
	Small city (up to 10,000 residents)	242	18
	Large city (over 10,000 residents)	988	73
Religion	Agnostic, Atheist	338	25
	Practising Catholic	586	44
	Non-practising Catholic	382	28
	Other	42	3
Year of study	1	232	17
	2	469	35
	3	345	25
	4	119	9
	5	105	8
	6	78	6
University	PMU	362	27
	GUM	328	24
	UMP	658	49

n—the number of respondents; %—relative frequency.

**Table 2:** Experience and opinions of the respondents on organ transplantation.

Question	Yes		No	
	n	%	n	%
Has any of your family members needed an organ transplantation?	69	5	1278	95
Has any of your family members or friends donated organ for transplantation?	63	5	1282	95
Do you agree that in Poland the number of organ donors is sufficient to meet the demand?	28	3	1303	97
Have you ever been informed about the possibility of donating one’s organs and about transplantation?	1002	74	344	26
Would you consent to organ donation on behalf of a family member?	34	3	1308	97
Have you discussed with your family members the possibility of donating your organs and transplantation?	783	58	560	42
Have you discussed with your friends the possibility of donating your organs and transplantation?	984	73	360	27
Have you signed the declaration of consent to tissue and organs donation for transplantation after your death (organ donor card)?	418	31	930	69

n—the number of respondents; %—relative frequency.

**Table 3:** Opinions of the respondents’ relatives concerning organ donation.

Opinion	Yes, in Favour		Don’t Know		Yes, Against		Other	
	n	%	n	%	n	%	n	%
Do you know the opinion of your father concerning organ donation?	449	33	807	60	55	4	37	3
Do you know the opinion of your mother concerning organ donation?	634	47	585	44	98	7	31	2
When in a partnership—do you know the opinion of your partner concerning organ donation?	420	31	272	20	34	3	622	46

n—the number of respondents; %—relative frequency.

**Table 4:** The effect of selected variables on the answer to the question “Would you consider donating your organs after death?”.

Variables		Yes		No		Have Doubts		F	*p* *
		M	SD	M	SD	M	SD		
Age		22.07	2.12	22.52	1.77	21.89	2.17	1.173	0.310
Variables		n	%	n	%	n	%	χ^2^	*p* *
Sex	Female	729	89.34	15	1.84	72	8.82	5.888	0.053
	Male	441	84.97	16	3.08	62	11.95		
Place of residence	Countryside	100	85.47	2	1.71	15	12.82	11.882	0.018
	Small city (up to 10,000)	196	81.67	8	3.33	36	15.00		
	Large city (over 10,000)	874	89.37	21	2.15	83	8.49		
Religion	Agnostic, atheist	312	93.69	5	1.50	16	4.80	19.877	0.003
	Practising Catholic	486	83.79	17	2.93	77	13.28		
	Non-practising Catholic	335	88.16	7	1.84	38	10.00		
	Other	24	85.71	1	3.57	3	10.71		
Year of studies	1	195	85.53	4	1.75	29	12.72	9.081	0.524
	2	406	86.75	11	2.35	51	10.90		
	3	302	88.56	7	2.05	32	9.38		
	4	108	92.31	2	1.71	7	5.98		
	5	93	88.57	5	4.76	7	6.67		
	6	66	86.84	2	2.63	8	10.53		
University	PMU	310	86.83	11	3.08	36	10.08	2.938	0.568
	GUM	287	88.31	9	2.77	29	8.92		
	UMP	573	87.75	11	1.68	69	10.57		

n—the number of respondents; %—relative frequency; *p* *—Pearson chi^2^ test.

**Table 5:** The effect of selected variables on the answer to the question: “Would you consider donating your organs after death?”.

Variables		Would You Consider Donating Your Organs after Death?							
		Yes		No		Have Doubts		χ^2^	*p* *
		n	%	n	%	n	%		
Given that we have two kidneys, therefore living kidney donation is possible, would you donate one kidney?	Yes, I would	480	92	6	1	36	7	40.474	<0.001
	No, I would not	90	76	11	9	17	15		
	Have doubts	595	86	14	2	81	12		
In your opinion, what is the risk of living kidney donation?	Great	42	84	5	10	3	6	27.984	<0.001
	High	340	87	5	1	48	12		
	Average	632	89	15	2	64	9		
	No risk	82	91	4	5	4	4		
	Don’t know	70	81	2	2	15	17		
If one of your family members (parents, children, siblings) needed a kidney transplant, would you donate your kidney?	Yes, I would	1095	89	23	2	111	9	48.258	<0.001
	No, I would not	10	67	3	20	2	13		
	Have doubts	61	70	5	6	21	24		
If you suffered from kidney disease and needed a kidney transplant, would you consent to receiving a kidney from a family member or would you rather be waitlisted and receive a kidney from a non-relative?	Yes, I would consent	858	89	19	2	92	9	7.817	0.099
	No, I would rather be waitlisted	80	90	4	4	5	6		
	Have doubts	232	84	8	3	36	13		

n—the number of respondents; %—relative frequency, *p* *—Pearson chi^2^ test.

**Table 6:** The effect of selected variables on the answer to the question: “Would you consider donating your organs after death?”.

Variables		Would You Consider Donating Your Organs after Death?							
		Yes		No		Have Doubts		χ^2^	*p* *
		n	%	n	%	n	%		
You only have one liver yet nowadays it is possible to donate a part of your liver to waitlisted patients. Would you donate a part of your liver?	Yes, I would	551	92	5	1	42	7	36.473	<0.001
	No, I would not	155	82	13	7	20	11		
	Have doubts	456	84	13	3	72	13		
What, in your opinion, is the risk of living liver donation?	High Risk	61	80	4	5	11	15	18.737	0.016
	Quite A Risk	260	86	9	3	34	11		
	Partial Risk	583	91	7	1	52	8		
	No Risk/Small Risk	148	87	6	4	16	9		
	Don’t Know	112	82	5	4	20	14		
If one of your family members (parents, children, and siblings) needed a liver transplant, would you donate a part of your liver?	Yes, I would	1095	89	24	2	111	9	79.367	<0.001
	No, I would not	6	55	4	36	1	9		
	Have doubts	61	72	3	3	21	25		
If you suffered from liver disease and needed a liver transplant, would you consent to receiving a liver from a family member or would you rather be waitlisted and receive the organ from a non-relative?	Yes, I would consent	904	90	19	2	81	8	22.395	<0.001
	No, I would rather be waitlisted	66	86	3	4	8	10		
	Have doubts	195	79	9	4	42	17		

n—the number of respondents; %—relative frequency, *p* *—Pearson chi^2^ test.

## Data Availability

Not applicable.

## Supplementary Materials

The following supporting information can be downloaded at: https://www.mdpi.com/article/10.3390/ijerph20043711/s1. Table S1: Sources of information on organ transplantation.
