# Supplementary material for: A Multicentre Study of the Attitude of Medical Students towards Organ Donation and Transplantation in Poland
Source: Int J Environ Res Public Health. 2023 Feb 20;20(4):3711. doi: 10.3390/ijerph20043711 (PMC9961742; doi:10.3390/ijerph20043711)
Supplement: Supplementary file 1 [file ijerph-20-03711-s001.zip › ijerph-2195234-supplementary.pdf]

Table S1. Sources of opinions regarding organ transplantation.

Sources of opinions regarding organ transplantation	Yes, positive		yes, negative		No		no answer	
	n	%	n	%	n	%	n	%
TV	901	67	33	2	385	29	29	2
Radio	347	26	20	1	900	67	81	6
Books, brochures	960	71	16	1	329	25	43	3
Magazines, newspapers	654	49	40	3	590	44	64	4
Movies	786	58	60	4	440	33	62	5
Friends	893	66	48	4	353	26	54	4
Family	610	45	82	6	589	44	67	5
Billboards, boards	578	43	17	1	690	51	63	5
Doctors, nurses	807	60	7	1	491	36	43	3
School	925	69	19	1	368	27	36	3
Lectures in other centers	361	27	10	1	895	66	82	6
Internet	1033	77	76	6	205	15	34	2
social media	720	53	60	4	495	37	73	6
Religious sources, e.g. the Church	195	14	130	10	940	70	83	6

N—the number of respondents; %—relative frequency.
